# Neuromuscular Coordination Deficit Persists 12 Months after ACL Reconstruction But Can Be Modulated by 6 Weeks of Kettlebell Training: A Case Study in Women's Elite Soccer

**DOI:** 10.1155/2017/4269575

**Published:** 2017-01-18

**Authors:** Mette K. Zebis, Christoffer H. Andersen, Jesper Bencke, Christina Ørntoft, Connie Linnebjerg, Per Hölmich, Kristian Thorborg, Per Aagaard, Lars L. Andersen

**Affiliations:** ^1^Department of Physiotherapy and Occupational Therapy, Faculty of Health and Technology, Metropolitan University College, Copenhagen N, Denmark; ^2^Human Movement Analysis Laboratory, Copenhagen University Hospital, Amager-Hvidovre, Copenhagen, Denmark; ^3^Department of Sports Science and Clinical Biomechanics, SDU Sport and Health Sciences Cluster (SHSC), University of Southern Denmark, Odense M, Denmark; ^4^Clinic of Sports Medicine, Danish Elite Sports Organization Team Denmark, Copenhagen, Denmark; ^5^Sports Orthopedic Research Center-Copenhagen, Arthroscopic Center, Department of Orthopaedic Surgery, Copenhagen University Hospital, Amager-Hvidovre, Copenhagen, Denmark; ^6^Institute of Sports Science and Clinical Biomechanics, University of Southern Denmark, Odense, Denmark; ^7^National Research Centre for the Working Environment, Copenhagen, Denmark; ^8^Physical Activity and Human Performance, Center for Sensory-Motor Interaction, Department of Health Science and Technology, Aalborg University, Aalborg, Denmark

## Abstract

The aim of the present single-case study was to investigate the effect of 6 weeks' kettlebell training on the neuromuscular risk profile for ACL injury in a high-risk athlete returning to sport after ACL reconstruction. A female elite soccer player (age 21 years) with no previous history of ACL injury went through neuromuscular screening as measured by EMG preactivity of vastus lateralis and semitendinosus during a standardized sidecutting maneuver. Subsequently, the player experienced a noncontact ACL injury. The player was screened again following postreconstruction rehabilitation, then underwent 6-week kettlebell training, and was subsequently screened again at 6-week follow-up. Prior to and after postreconstruction rehabilitation the player demonstrated a neuromuscular profile during sidecutting known to increase the risk for noncontact ACL injury, that is, reduced EMG preactivity for semitendinosus and elevated EMG preactivity for vastus lateralis. Subsequently, the 6-week kettlebell training increased semitendinosus muscle preactivity during sidecutting by 38 percentage points to a level equivalent to a neuromuscular low-risk profile. An ACL rehabilitated female athlete with a high-risk neuromuscular profile changed to low-risk in response to 6 weeks of kettlebell training. Thus, short-term kettlebell exercise with documented high levels of medial hamstring activation was found to transfer into high medial hamstring preactivation during a sidecutting maneuver.

## 1. Introduction

Anterior cruciate ligament (ACL) injuries in sports are of increasing concern for physicians and scientists worldwide [1]. In United States, between 100.000 and 300.000 ACL reconstructions are performed every year [2]. The enigmatic phenomenon “noncontact ACL injury” in sports has been objective for increasing preventative scientific research efforts in recent years. Across sports, noncontact ACL injuries account for about 60% of all ACL injuries registered [3]. A noncontact ACL injury is defined as an ACL injury sustained by the athlete without extrinsic contact to another player or object on the field [4]. Thus, the player performs a sports task, for example, a sidecutting maneuver, which has been executed numerous times before; only this time the ACL is ruptured. The mechanisms underlying this specific type of sports injury remain still in part a mystery, and identification of risk factors predisposing for noncontact ACL injury has high clinical relevance and priority in both injury prevention and rehabilitation therapy.

In the present case study, we report findings obtained in a 21-year-old female elite soccer player (Danish National Team), who sustained a noncontact ACL injury 3 years* after* being tested in our laboratory for neuromuscular performance during soccer relevant movements, including a sidecutting manoeuver. At the time of her ACL injury, we had identified a “high-risk” zone [5] for noncontact ACL injury based on a reduced EMG preactivity for m. semitendinosus (ST) and elevated EMG preactivity for m. vastus lateralis (VL), expressed as a large differential VL-ST EMG preactivity during standardized sidecutting maneuvers. The identification of a high-risk zone suggests that specific preventative efforts should be performed for players identified within this high-risk zone. Thus, we examined in retrospect the initial sidecutting test performed and found that the player displayed a neuromuscular high-risk profile for noncontact ACL injury as defined by Zebis et al. (2009) [5], that is, characterized by low semitendinosus (ST) EMG preactivity in combination with high vastus lateralis (VL) EMG preactivity. Thus, this case report represents an exclusive possibility of studying the plasticity of a motor program executed during a high-risk movement (sidecutting), from “prior to ACL injury” to “after ACL reconstruction and rehabilitation” and, finally, following specific neuromuscular training targeting the single most important ACL agonist, that is, the medial hamstring muscle (ST).

The aim of the present case study was to describe the effect of 6 weeks' kettlebell training on the neuromuscular profile for noncontact ACL injury during sidecutting in a high-risk elite athlete returning to sport after ACL reconstruction.

## 2. Case Presentation

*August 2006*. A 21-year-old female soccer player competing at elite level (Table 1) and with no previous history of knee injuries was engaged in a study examining functional performance during selected sports tasks. Among other tests, neuromuscular hamstring and quadriceps EMG preactivity was obtained during a standardized sidecutting maneuver. In 2009, a study identified a neuromuscular high-risk profile for noncontact ACL injury during sidecutting [5]. 

*May 2009*. The player sustains a documented (video recorded) noncontact ACL injury in the right knee during match play. Retrospectively examined, the initial test (2006) revealed that the female soccer player, in her uninjured state, displayed a high-risk profile during sidecutting consistent with the study reports by Zebis et al. (2009) [5]. 

*June 2009*. Surgical ACL reconstruction is performed using a semitendinosus-gracilis autograft. 

*April 2010*. After 10 months of standardized rehabilitation (more details provided below), the player is deemed fully rehabilitated by medical professionals to return to preinjury sports activities, and the player returns to soccer at elite level. 

*May 2010*. The player is involved in elite soccer at her preinjury level (Table 1). A second test for neuromuscular preactivity during a standardized sidecutting maneuver is performed. At this point in time, the player persists to display markedly reduced ST EMG preactivity during the sidecutting maneuver. Consequently, a 6-week training program involving kettlebell swing exercise is introduced to the player. 

*July 2010*. A final third test involving neuromuscular preactivity assessment during a standardized sidecutting maneuver is performed in our lab after completion of the 6 weeks' kettlebell intervention. 

In August 2011, the player sustained an ACL rerupture due to a traumatic contact situation during match play. The screening model used in the present study solely addresses the risk of noncontact ACL injury [5]. Thus, contact injuries are not accounted for in this model.

Written informed consent was obtained from the athlete prior to all testing and analyses.

## 3. Test Protocol

### 3.1. The Sidecutting Maneuver

The subject was screened for neuromuscular EMG preactivity while performing a standardized sidecutting maneuver in the laboratory. A previous study has demonstrated high test-retest reproducibility for magnitude and timing of the EMG activity during sidecutting [6] showing that this maneuver represents a consistent motor program in the CNS of trained players. In support of this notion, the sidecutting maneuver has been found to remain unchanged during a regular season with training and match play [6].

### 3.2. Neuromuscular Screening by EMG Recording

Surface EMG electrodes were placed on the preferred push-off leg (i.e., left leg) on vastus lateralis (VL), biceps femoris (BF), and semitendinosus (ST) muscles according to recommended standard procedures [7]. For detailed description of the laboratory setup see Zebis et al. (2009) [5].

During later offline analysis all EMG signals were high-pass filtered at a 5 Hz cutoff frequency (4th-order zero-lag Butterworth filter) and subsequently smoothed by a symmetrical moving RMS filter of 30 ms [8]. Mean RMS EMG amplitude was obtained for all muscles examined instantly before ground contact, defined as 0–10 ms prior to foot strike on the force plate, and subsequently normalized to the peak RMS EMG amplitude recorded during the sidecutting maneuver [6]. The average of 3 trials was calculated for each muscle in each test.

### 3.3. Countermovement Jump (CMJ)

CMJ measurements were performed on a force plate (AMTI, Advanced Mechanical Technology, Inc.). Countermovement jumping was performed with the hands placed at the hip (akimbo), and maximal jump height was calculated by time-integration (0.001 time constant) of vertical ground reaction force as previously described by Caserotti et al. (2001) [9].

## 4. Rehabilitation after ACL Reconstruction

A standardized rehabilitation program, using generally accepted progression criteria was followed [10]. The rehabilitation protocol included specific goals for range of motion, muscle function, and functional performance, and these goals had to be met before the player could progress to the next level [10]. The player was supervised weekly during the entire postoperative rehabilitation phase by a trained physiotherapist.

In the first 12 weeks of rehabilitation, seated knee extension, squat on one leg, squat on two legs, heel raise, and standing on one leg were part of the training program, all exercises performed in a slow controlled manner. These exercises have previously been evaluated by EMG recording in ACL patients, 5 weeks after ACL reconstruction, demonstrating hamstring muscles activity levels corresponding to 19%–68% of MVC EMG [11]. The balance/coordination exercises during the first 12 weeks of rehabilitation included two-legged standing on wobble board and one-legged stance on balance mat, which previously has been reported to activate the hamstring muscles at low EMG activity levels [12].

A detailed description of the first 12 weeks of rehabilitation is presented in Table 2.

After 12 weeks of postoperative rehabilitation, strength training was progressively increased and open kinetic chain exercise, that is, loaded knee extension in machine, as well as free-weight barbell squat was included in the rehabilitation program. The majority of strength training exercises in the rehabilitation program were aimed at targeting the quadriceps muscles, with the hamstring muscles contracting actively as antagonists (knee extension) or synergists (hip extension). In a previous study, relatively low levels of ST EMG coactivation were observed during isolated knee extension exercise, free-weight squat, and seated leg press (9% to 22% of max EMG) in young healthy males [13]. Further, a preferential recruitment of the lateral hamstring muscle, biceps femoris (BF), over the medial hamstrings (ST) was noted during these exercises [13]. A single isolated strength training exercise for the hamstring muscles (prone leg curl exercise) was included in the rehabilitation protocol, which has been reported to involve high and comparable levels of ST and BF EMG muscle activities (>60% of max EMG) [13, 14]. After 4 months, higher level balance/coordination exercises on stable and unstable surface were included in the rehabilitation program. After 6 months, one-legged jump, landing, cutting, pivoting, and running drills were progressively included in the rehabilitation program [10]. However, a complete evidence-based neuromuscular training program was not followed by the player, for example, the program described by Myklebust et al., 2003 [15]. The ballistic balance/coordination exercises (i.e., one-legged jumps on different surfaces) have previously been evaluated in female elite athletes, where ST EMG activity levels were reported to range from 44% to 65% of MVC EMG [14]. After week 35, full soccer training was attended and at week 40 the player was engaged in full competition.

## 5. Return-to-Play

Upon completion of the postoperative rehabilitation phase, the player was tested according to the recommendations of return-to-play [16]. The player performed 3 single-legged hop tests: the single hop for distance, triple hop for distance, and single vertical hop. These tests have demonstrated good test-retest reliability in normal, young adults [17] and in patients after ACL reconstruction [18]. Finally, the player's self-reported knee function was scored by a validated questionnaire [19]. In April 2010, the player reached a level of the injured leg corresponding to ≥100% of the uninjured leg in all tests, and the player was cleared ready for return-to-play by the medical staff (PT, MD).

## 6. Kettlebell Swing Exercise Intervention

Since neuromuscular testing revealed that the players ST activation deficit persisted to exist during sidecutting after completion of the standard rehabilitation protocol, it was decided that the player should perform 6 weeks of additional training that was designed to preferentially target the medial hamstring muscle (more details given below). In this context, the kettlebell swing exercise has been reported to be particularly effective to targeting the ST muscle, demonstrating markedly higher EMG activity levels than ballistic one-legged balance/coordination exercises [14]. The kettlebell swing is a ballistic exercise performed standing on two legs where the highest external load is when the hamstring muscles are most stretched, that is, hip flexed and knee near straight. At this point, the knee joint position resembles the knee joint angle observed in a typical noncontact ACL injury situation [20]. Due to the stable two-legged position when swinging the kettlebell, the exercise is considered a safe exercise with respect to the knee joint. The kettlebell exercise intervention was initiated at the time when the player had returned to her preinjury activity level (May 2010, Table 1).

**Figure 1:**
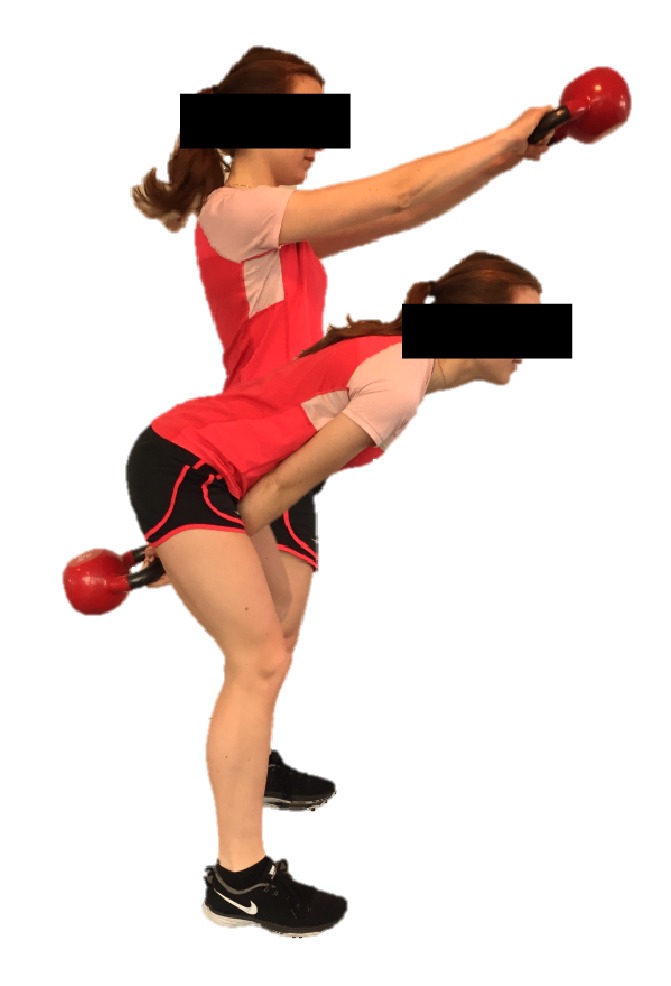
An illustration of the kettlebell swing exercise.

The player performed kettlebell swings using a 16 kg kettlebell according to the strength level of the subject and progressed later to 20 kg at the end of the intervention period. The kettlebell weight was chosen to match a weight where the subject was able to swing 20 times using proper technique. In this order, the kettlebell exercise has been shown to preferentially activate the medial hamstring muscle and at high EMG activity levels [14]. The player was supervised in proper technique by an educated kettlebell instructor. Kettlebell exercise was performed by forcefully swinging the kettlebell back between the legs by flexing the hips and keeping the knees slightly flexed (~10–15°) and to quickly reverse the direction with an explosive extension of the hips, swinging the kettlebell out to chest level where the hips and knees are extended and the subject is standing upright [14] (Figure 1).

In total, the player performed 10 training sessions during the 6-week period. Each training session consisted of 3–5 sets, with each single set corresponding to 20 swings and 20 seconds' pause in a 2-minute time interval. In sessions 1–4, kettlebell swings were performed using a 16 kg kettlebell. In sessions 5–10, kettlebell swings were performed with a 20 kg kettlebell.

## 7. Results

### 7.1. Test I: Neuromuscular Coordination Assessment

Prior to ACL injury the player demonstrated a neuromuscular pattern during sidecutting known to increase the risk for noncontact ACL injury, that is, reduced EMG preactivity for the ST muscle (21% of max EMG, Table 3) and elevated EMG preactivity for the VL (i.e., VL-ST EMG preactivity difference ≥ 33%) [5].

### 7.2. Test II: Neuromuscular Coordination Assessment

Ten months after ACL reconstruction (including standardized postsurgical rehabilitation) the player was deemed ready to return to play by the medical staff (PT, MD). However, the player demonstrated a persisting pattern of high-risk neuromuscular activity during the sidecutting maneuver, characterized by reduced ST preactivity (23% of max EMG, Table 3). Six weeks of kettlebell training was therefore initiated, based on previous observation that kettlebell swing induces preferential high levels of semitendinosus EMG activity during execution [14].

### 7.3. Test III: Neuromuscular Coordination Assessment

After 6-week kettlebell training, the player increased ST preactivity during sidecutting from 23% to 61% of max EMG (Table 3), that is, increasing ST preactivity by 38 percentage points, consequently defined as a low-risk profile (VL-ST preactivity difference < 33%) [5]. In contrast, the lateral hamstring muscle (BF) displayed constancy in preactivity levels (17–26% of max EMG) across all time points examined (Table 3).

### 7.4. Countermovement Jump (CMJ)

No change in functional performance, measured as maximal CMJ height, was observed throughout the study period (Table 4).

## 8. Discussion

In the present case study, a female elite soccer player was identified in retrospect with a high-risk neuromuscular profile [5] prior to sustaining a noncontact ACL rupture for the first time. Despite ACL reconstruction and subsequent period of standardized rehabilitation [10], the player continued to display a neuromuscular high-risk profile by the time of return-to-sport.

Although high quadriceps activity also seems to predispose for future ACL rupture, high knee extensor activity is essential to gain power and speed in explosive movements as the sidecutting maneuver. Thus, the main focus in the present case was to implement training that targeted an upregulation in medial hamstring (i.e., ST) muscle activity, for which reason the kettlebell exercise was chosen. Notably, six weeks of kettlebell training was effective for changing the neuromuscular profile during sidecutting from high- to low-risk behavior, due to an increased ST EMG preactivity during the sidecutting maneuver.

Excessive dynamic knee joint valgus moment during drop jumping has previously been identified to predispose for ACL injury [21]. The medial hamstring muscles are theoretically the only major muscles to actively counteract dynamic valgus [22]. In support of the medial ST muscle as the single most important ACL agonist, we have previously identified a “high-risk” zone for noncontact ACL injury comprised by low ST preactivity in concurrence with high VL preactivity during sidecutting [5]. In the present “high-risk” case, preventative efforts were not initiated prior to the athlete's first noncontact ACL injury because data from the screening study that established this risk profile had not been finally collected and analyzed. At the time of ACL injury and the subsequent rehabilitation, the neuromuscular screening method and ACL injury risk profile used in the present case study had been established and published [5]. Despite the fact that all standard procedures for ACL surgery, rehabilitation, and safe return-to-play were followed, the player persisted to display a high-risk profile characterized by low ST preactivity level during sidecutting. This finding indicates that standard rehabilitation did not sufficiently target the neuromuscular high-risk profile that the player displayed prior to the first-time ACL injury. In other words, the player appeared to remain at high-risk of noncontact ACL injury despite being discharged from rehabilitation and cleared to play.

We have previously reported that a multiexercise neuromuscular training program known to reduce the incidence of ACL injury among female elite athletes [15] may achieve this through a selective upregulation in ST preactivity during sidecutting [6]. In the present case report, various postural balance and intermuscular coordination exercises from the neuromuscular program [15] were introduced in the rehabilitation phase. Despite the implementation of such specific ACL injury prevention exercises, the player still demonstrated a high-risk profile at the time of “return-to-play.” This indicates that a more aggressive training approach should be implemented when seeking to evoke a change in an established and consistent motor program. Thus, we focused exclusively on the ability to activate the semitendinosus and introduced an intervention with a single exercise, that is, kettlebell swing, known to preferentially activate the semitendinosus and at high activation levels [14].

The intervention strategy with six weeks of kettlebell training changed the neuromuscular profile from high- to low-risk [5]. Thus, the present case study indicates that a specific single exercise intervention targeting the ST muscle may be able to remodulate an existing motor program executed during a movement associated with noncontact ACL injury.

As seen in the present study participant, ACL ruptures typically are reconstructed by using the ST tendon as an autograft [23]. A follow-up MRI of the player in 2011 confirmed regeneration of the ST tendon. In line, a previous review reports that regeneration of the semitendinosus tendon may be confirmed in the majority of ACL patients reconstructed by harvesting of the ST tendon [24]. However, the volume of the semitendinosus muscle in the reconstructed limb has been reported to remain reduced compared to that of the uninjured limb [25]. Thus, in respect to dynamic knee joint control during sports activities involving cutting and pivoting tasks, it could be speculated whether ST graft harvesting actually leads to an elevated risk for sustaining secondary ACL injury (rerupture). In fact, recent data reveals that the revision rate after using hamstring tendon autograft reconstruction is higher compared with patella tendon autograft reconstruction, especially among the youngest patient group [26, 27]. Thus, introducing ballistic exercise modalities such as the kettlebell swing with preferentially high levels of medial hamstring activation seems essential, in both primary and secondary ACL injury preventative strategies.

In conclusion, this case describes an elite female soccer player who, after sustaining a noncontact ACL injury, having the ACL reconstructed and fulfilling 10 months of intensive ACL rehabilitation, still had a high-risk neuromuscular profile as she had preinjury. After only 6 weeks of kettlebell training, the neuromuscular pattern during sidecutting reversed from high- to low-risk. Thus, this exercise modality might be an important supplement to the standardized rehabilitation among patients aiming to return to sports activities. Future large-scale prospective studies should confirm whether the present findings can be used in the primary and secondary prevention of noncontact ACL injuries.

## Figures and Tables

**Table 1:** Physical activity scheme at time of testing.

Components	Test I: Before noncontact ACL injury	Test II: After surgery & rehabilitation	Test III: After kettlebell training
Soccer practice 90 min (/wk)	4-5	4-5	4-5
Match play 90 min (/wk)	1	1	1
Strength training (/wk)	2	2	2
Squat	3 × 8–10 RM	3 × 6 RM	3 × 6 RM
Leg press	3 × 4–6 RM	3 × 10 RM	3 × 10 RM
Lateral raise	—	3 × 10 RM	3 × 10 RM
Nordic hamstring	3 × 10 reps	—	—
Knee extension	3 × 8–10 RM	3 × 10 RM	3 × 10 RM
Leg curl	3 × 8–10 RM	3 × 10 RM	3 × 10 RM
Kettlebell	—	—	See description

**Table 2:** Postoperative rehabilitation program.

Components	0–2 weeks	3–6 weeks	7–9 weeks	10–12 weeks
Soccer practice 90 min (/wk)	—	—	—	
Match play 90 min (/wk)	—	—	—	
Strength training (/wk)	—	—	—	3-4
Free-weight squat with barbell	—	—	—	—
Seated leg press (two-leg)	—	—	—	1 × 15 reps
Seated leg press (one-leg)	—	—	—	3 × 10 reps
Rotary calf	—	—	—	3 × 10 reps
Standing calf	—	—	—	3 × 10 reps
Knee extension	—	—	—	—
Prone leg curl	—			3 × 10 reps
Bicycling (/wk)	—	7	7	7
Exercises: unloaded (/wk)	7	7	7	7
Hip flexion/extension		3 × 10 reps	3 × 10 reps	3 × 10 reps
Knee flexion/extension		3 × 10 reps	3 × 10 reps	3 × 10 reps
Supine pelvic lifts (two-leg)		3 × 10 reps	3 × 10 reps	3 × 10 reps
Supine pelvic lifts (one-leg)		2 × 10 reps	2 × 10 reps	2 × 10 reps
Hip adduction (lying)		3 × 10 reps	3 × 10 reps	
Hip abduction (lying)		3 × 10 reps	3 × 10 reps	
Forward lunges		3 × 10 reps	3 × 10 reps	3 × 10 reps
Walking backwards		3 × 20 steps	3 × 20 steps	
Heel lifts		3 × 10 reps	3 × 10 reps	3 × 10 reps
Prone straight leg lift		3 × 10 reps	3 × 10 reps	
One-leg squat		—	3 × 10 reps	3 × 10 reps
Two-leg squat		—	3 × 10 reps	3 × 10 reps
Supine straight leg lift		—	3 × 10 reps	
One-leg standing		1 × 5 min	1 × 5 min	—
Exercises: equipment (/wk)	—	7	7	7
Hip abduction (elastics)		—	3 × 10 reps	3 × 10 reps
Prone knee flexion (elastics)		—	—	3 × 10 reps
Two-leg standing (wobble)		—	1 × 5 min	1 × 5 min
One-leg standing (mat)		—	—	3 × 10 reps
Step up (box)		—	2 × 10 reps	3 × 10 reps
Stretching exercises (/wk)		7	7	7
Quadriceps muscles		40 sec	40 sec	40 sec
Hamstring muscles		40 sec	40 sec	40 sec
Calf muscles		40 sec	40 sec	40 sec

**Table 3:** Normalized EMG preactivity during sidecutting.

Preactivity	Test I: Before noncontact ACL injury	Test II: After surgery & rehabilitation	Test III: After kettlebell training
Semitendinosus (% of max EMG)	21%	23%	61%
Biceps femoris (% of max EMG)	23%	26%	17%

**Table 4:** Countermovement jump (CMJ) performance.

Height	Test I: Before noncontact ACL injury	Test II: After surgery & rehabilitation	Test III: After kettlebell training
CMJ (cm)	31.9	31.3	31.6
